# Intrathecal Fentanyl Pump Placement in a Patient With Chronic Pain Caused by Hereditary Multiple Exostoses: A Case Report

**DOI:** 10.7759/cureus.73240

**Published:** 2024-11-07

**Authors:** Hayden Tageant, Corrie N Jackson, Giustino Varrassi, Alberto Pasqualucci, Sahar Shekoohi, Alan D Kaye

**Affiliations:** 1 School of Medicine, Louisiana State University Health Sciences Center, Shreveport, USA; 2 Pain Medicine, Fondazione Paolo Procacci, Rome, ITA; 3 Anesthesia and Critical Care, University of Perugia, Perugia, ITA; 4 Anesthesiology, Louisiana State University Health Sciences Center, Shreveport, USA

**Keywords:** chronic pain management, fentanyl, hereditary multiple exostoses, intrathecal pump, multiple osteochondromas, pain

## Abstract

Hereditary multiple exostoses (HME) is a genetic disorder defined by the formation of benign bone tumors known as exostoses, which can lead to chronic pain and functional impairment. This case report details a 57-year-old man with a long-standing history of severe diffuse bone pain attributed to HME. Despite various treatments, his pain remained poorly controlled until an intrathecal pump with fentanyl was implanted. This intervention significantly improved his pain management and overall quality of life. The manuscript explores clinical presentation, diagnostic approach, and treatment outcomes, highlighting the challenges and benefits of intrathecal drug delivery systems in managing refractory pain associated with HME.

## Introduction

Hereditary multiple exostoses (HME) or hereditary multiple osteochondromas (HMO), is a genetic disorder typically inherited in an autosomal dominant pattern. Characterized by the development of multiple benign bone tumors, HME can result in significant musculoskeletal pain and functional limitations related to deformities and bone growths that affect various parts of the skeleton [1,2]. HME is a rare condition with a reported prevalence of approximately 1 in 50,000 in Caucasians [1]. The condition leads to debilitating chronic pain, mobility issues, and other complications that severely decrease the quality of life [3,4]. Pain management in HME is complex and related to the widespread nature of exostoses and variability in pain localization and intensity [5,6]. Traditional pain management strategies, including oral medications and physical therapy, often provide inadequate relief for severe cases [7]. Intrathecal drug delivery systems, such as intrathecal pumps, offer a promising alternative by delivering pain medications directly into the cerebrospinal fluid, thus potentially enhancing efficacy and minimizing systemic side effects [8-10].

HME follows an autosomal dominant inheritance pattern, with mutations in the EXT1 or EXT2 genes leading to the formation of multiple osteochondromas [11]. This genetic basis explains why the condition appears within families and manifests in various generations. In this case, the patient and several family members, including his children, were affected [12]. 

This case report presents a 57-year-old man with HME who experienced debilitating pain limiting his ability to walk and complete basic daily tasks, despite multiple conservative treatments including analgesic and non-analgesic medications, physical therapy, and surgeries. His pain management improved significantly following the implantation of an intrathecal pump with fentanyl.

## Case presentation

Clinical presentation

**Figure 1:**
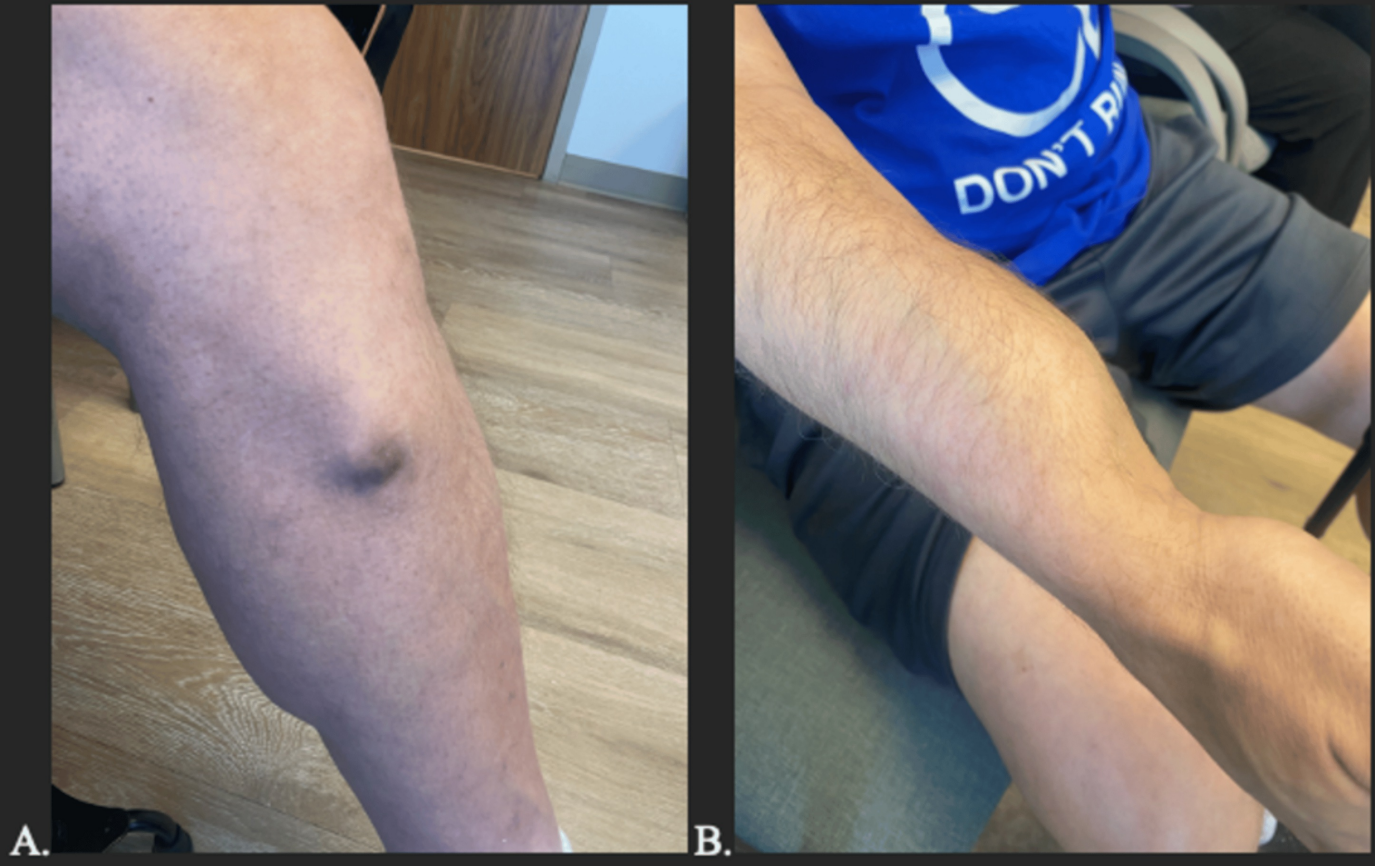
Bone masses in the patient with HME, illustrating (A) the left lower extremity and (B) the right arm as examples of bone lesions associated with the condition. HME: Hereditary Multiple Exostoses

The patient was a 57-year-old man with a diagnosis of HME made at the age of five. His symptoms began with diffuse bone pain, primarily affecting his legs but eventually encompassing his entire body. The patient had over 40 bone masses, most in his lower extremities (Figure 1).

His pain was mainly located at the bone mass sites. It was described as aching, burning, crushing, dull, sharp, shooting, stabbing, tight band, and tingling, with intensity ranging from 5/10 on the best to 10/10 on the worst day. The pain worsened at night, and the masses were tender to palpation. It interfered significantly with daily activities, sleep, and work and was exacerbated by almost all activities and positions.

Initial treatments included a four-month trial of Norco 10 mg oral pain medication and physical therapy, but these provided only partial and temporary relief. The patient continued to suffer from severe pain, which was especially unrelenting at night. After discussing the risks and benefits of oral medication for chronic pain with the patient, we decided on a trial with an intrathecal pump. A trial using morphine 0.25 mg failed and resulted in significant urinary retention. A subsequent intrathecal opioid trial with fentanyl in a dose of 20 mcg proved highly effective, leading to a decision to implant it permanently.

Diagnosis of Hereditary Multiple Exostoses

Clinical evaluation and imaging studies established the diagnosis of HME. The patient's history of bone masses and persistent pain, along with his family history (including his sister and two of his children), supported the diagnosis. Genetic testing confirmed mutations associated with HME, though specific mutations were not detailed in this case [1].

Treatment and Procedure

After obtaining informed consent, the patient underwent an intrathecal pump implantation procedure on May 30, 2024. General anesthesia was administered, and the patient was positioned in the left lateral decubitus position. A time-out was performed to confirm the patient’s identity, procedure, and site prior to induction of general anesthesia.

A Medtronic introducer needle kit was used to access the L2-L3 interlaminar interspace using fluoroscopy, and cerebrospinal fluid (CSF) was confirmed to ensure proper catheter placement. A Medtronic soft tip catheter was advanced to the T8 level since most of the pain was in his lower extremities, and its position was verified using fluoroscopy. A pocket was created in the right lower quadrant for the Medtronic SynchroMed III pump, which was filled with 20 ml of fentanyl (50 mcg/ml) at a rate of 8 mcg/24 hours. The catheter was connected to the pump, and the system was secured with a multi-layer closure.

Postoperatively, the patient was monitored for respiratory depression and other potential complications overnight in the hospital. He was discharged the following day and reported significant improvement in his pain, particularly in his lower extremities, with a decrease in pain levels from 8/10 to 4/10, with a further decrease noted with increasing doses of the intrathecal fentanyl administration.

Follow-up

The patient has been coming for regular follow-ups. He has had a recent pump refill and is doing well with a high level of comfort. The intrathecal pump was checked via a Bluetooth tablet at every visit to check the rate and amount of fentanyl left in the system. The patient is currently on 10.5 mcg/24 hours and has a pain score of 3-4/10 with increased activity and improved quality of life.

## Discussion

In this case, the intrathecal pump with fentanyl provided substantial improvement in pain management compared to previous treatments [13]. Fentanyl, an opioid 80-100 times more potent than morphine, was chosen for the intrathecal pump due to its efficacy in providing pain relief while minimizing the required dose compared to systemic opioids [14-16]. It was considered since the patient had previously tolerated this analgesic. The success of this intervention highlights the potential benefits of intrathecal drug delivery systems for patients with severe, refractory pain [17]. The patient reported significant improvements in pain levels, from 8/10 to 4/10, and noted increased activity. There were further reductions in the pain score with increasing doses of fentanyl at an increase of 5-10% per visit every two weeks over two months, and significantly improved quality of life.

Intrathecal pumps are supported by evidence showing effectiveness in managing chronic pain, particularly in cases where other treatments have failed [18]. Studies have demonstrated that intrathecal drug delivery systems can provide long-term pain relief and improve functionality in patients with complex pain conditions [8,19-22]. In this case, the successful outcome underscores the importance of individualized treatment plans and the potential of intrathecal pumps in managing chronic pain associated with genetic disorders like HME [23]. This is the first case in the global literature of pain management for HME that describes the use of an intrathecal opioid delivery system.

## Conclusions

This case report provides a detailed account of refractory pain management in a patient with HME using an intrathecal drug delivery system. The positive outcomes observed with the intrathecal pump demonstrate its efficacy as a viable option for complex pain management in challenging cases, including pain mediated by HME. The report highlights the significant impact of intrathecal drug delivery systems in managing chronic pain states, including pain related to bony deformities due to congenital conditions. Despite extensive conservative management, the patient’s pain remained severe and debilitating until the implantation of an intrathecal pump with fentanyl. This intervention provided substantial relief and improved the patient’s quality of life, illustrating the effectiveness of targeted pain management strategies and the role of this approach in broader applications related to complex and refractory pain conditions. Further research is warranted to explore long-term outcomes with intrathecal drug delivery systems and optimize treatment approaches for patients with similar conditions.
